# Thoracic limb morphology of the red panda (*Ailurus fulgens*) evidenced by osteology and radiography

**DOI:** 10.4102/ojvr.v82i1.953

**Published:** 2015-07-15

**Authors:** Modesta Makungu, Hermanus B. Groenewald, Wencke M. du Plessis, Michelle Barrows, Katja N. Koeppel

**Affiliations:** 1 Department of Anatomy and Physiology, University of Pretoria, South Africa; 2 Department of Veterinary Surgery and Theriogenology, Sokoine University of Agriculture, Tanzania; 3 Ross University School of Veterinary Medicine, St. Kitts, West Indies; 4 Veterinary Services and Conservation Medicine, Bristol Zoo Gardens, United Kingdom; 5 Johannesburg Zoo, Johannesburg, South Africa

## Abstract

The red panda (*Ailurus fulgens*) is distributed primarily in the Himalayas and southern China. It is classified as a vulnerable species by the International Union for Conservation of Nature. The aim of this study was to describe the normal osteology and radiographic anatomy of the thoracic limb of the red panda. Radiography of the right thoracic limb was performed in seven captive adult red pandas. Radiographic findings were correlated with bone specimens from three adult animals. The scapula was wide craniocaudally and presented with a large area for the origin of the teres major muscle. The square-shaped major tubercle did not extend proximal to the head of the humerus. The medial epicondyle was prominent. A supracondylar foramen was present. The radial tuberosity and sesamoid bone for the abductor digiti I longus were prominent. The accessory carpal bone was directed palmarolaterally. Metacarpal bones were widely spread. The thoracic limb morphology of the red panda evidenced by osteology and radiography indicated flexibility of the thoracic limb joints and well-developed flexor and supinator muscles, which are important in arboreal quadrupedal locomotion. Knowledge gained during this study may prove useful in identifying skeletal material or remains and diagnosing musculoskeletal diseases and injuries of the thoracic limb.

## Introduction

The red panda (*Ailurus fulgens*) is classified as a vulnerable species by the International Union for Conservation of Nature (IUCN 2014). It is an arboreal animal commonly kept in zoological gardens.

The red panda is affected by musculoskeletal diseases and injuries similar to those reported in domestic cats and dogs and radiography is commonly used as the first diagnostic imaging modality. Diseases and injuries involving the musculoskeletal system of the thoracic limb, such as osteoarthritis, hyperostotic bone disease and fractures, have been reported in the red panda (Lynch, McCracken & Slocombe 2002; Philippa & Ramsay 2011; Preece 2011).

Knowledge of the normal osteology and radiographic anatomy is important for accurate interpretation and diagnosis of musculoskeletal diseases involving the thoracic limb. The normal osteology and radiographic anatomy of the thoracic limb in companion animals is well documented (Nickel *et al.* 1986; Smallwood & Spaulding 2013; Thrall & Robertson 2011), which serves as a reference for diagnosis of musculoskeletal diseases.

Little information is available on the osteology of the thoracic limb of the red panda (Carlsson 1925) and to our knowledge, the normal radiographic anatomy of the thoracic limb has not been described in this species. The aim of this study was to describe the morphology of the thoracic limb of the red panda evidenced by osteology and radiography as a reference for clinical use and identification of skeletal material and skeletal remains.

## Materials and methods

### Radiography

Radiography of the right thoracic limb was performed in seven intact, skeletally mature, adult captive red pandas belonging to the Johannesburg Zoo. Of the seven animals, five were male and two were female. The age of the animals ranged from 1.4 years to 14.3 years (mean 7.3 ± 5.4 years).The mean weight of the animals was 4.5 kg ± 0.7 kg (range: 3.7 kg − 5.6 kg). Radiography was performed under general anaesthesia during annual health examinations using a table top technique.

In six animals, an EVA-HF525 X-ray machine (Comed Medical Systems, Korea) was used at a source-to-image distance (SID) of 95 cm. An automatic X-ray film processor (model: CP-345; ELK Corporation, Japan) was used. Mammography films (UM-MA, Fujifilm, Japan) were used in combination with UM-MA screens (Fujifilm Europe, Germany). All radiographs were obtained using a peak kilovoltage (kVp) range of 46−50 and a charge of 5 mAs. Radiographic images were digitised using a digital camera (Canon 5DMARK2, Canon, Japan).

In one animal, radiography was performed using a Siemens Polymat 50 X-ray machine (Siemens, Germany) and images were obtained using a computed radiography system (Fuji Axim FCR Capsula XL, Fujifilm, Japan) at an SID of 100 cm. A kVp range of 48–60 and charge range of 4 mAs – 5 mAs were used.

Previous radiographs of the right thoracic limb of two red pandas, which were taken at the Bristol Zoo Gardens for clinical evaluation before skeletal maturity (10 and 7 months old, respectively), were retrieved and evaluated for the location of physes.

### Osteology

Bone specimens of skeletally mature adult animals (one from the National Museum of Scotland and two euthanised animals from the Johannesburg Zoo) were used for the osteology study. Photographs of bone specimens were obtained using a digital camera (Canon 5DMARK2, Canon, Japan). A report on the myology of the thoracic limb of the red panda (Fisher *et al.* 2009) was used to locate the sites for the origins, insertions and functions of the various muscles of the thoracic limb.

### Bone measurements

Bone measurements of the right thoracic limb were performed on radiographic images of skeletally mature captive red pandas. This excludes measurements for the scapula, which were performed on bone specimens. The maximum lengths of bones were measured from the proximal to the distal extremities. The maximum lengths of the humerus, radius and ulna were measured on the mediolateral (ML) view, whereas those of the metacarpals, phalanges and sesamoid bones were measured on the dorsopalmar (DPa) view of the manus.

The craniocaudal (CrCd) diameters of the humerus, radius and ulna were measured on the ML view at mid diaphysis. The ML diameter of the sesamoid bone for the abductor digiti I longus was measured on the DPa view of the manus as the maximum diameter perpendicular to the maximum length. Radiographic measurements were not compensated for magnification.

#### Statistical analysis

Statistical analysis was performed using the StatView statistical package (SAS Institute, USA). Mean, range and standard deviation (s.d.) were calculated. Data were expressed as mean ± s.d.

## Ethical considerations

This study was approved by the Animal Use and Care Committee of the University of Pretoria.

## Results

Measurements of bones described in the figures are shown in Tables 1–3.

**TABLE 1:** Measurements of the scapula, humerus, radius and ulna in captive red pandas.

Bone	Variables	Number of animals	Mean ± s.d. (cm)	Range (cm)
Scapula	Length^a^	3	7.00 ± 0.10	6.90−7.10
	Craniocaudal diameter^a^	3	6.32 ± 0.08	6.25−6.40
Humerus	Length^b^	5	11.58 ± 0.48	11.10−12.00
	Craniocaudal diameter^b^	5	1.12 ± 0.08	1.00−1.20
Radius	Length^b^	5	9.36 ± 0.93	8.20−10.80
	Craniocaudal diameter^b^	5	0.51 ± 0.02	0.50−0.55
Ulna	Length^b^	5	10.64 ± 0.99	9.00−11.50
	Craniocaudal diameter^b^	5	0.60 ± 0.07	0.50−0.70

s.d., standard deviation.

^^a^^, Measurements from bone specimens.

^^b^^, Radiographic measurements not compensated for magnification.

**TABLE 2:** Radiographic measurements of metacarpal bones and phalanges in captive red pandas.

Bone^a^	Mean length ± s.d. (cm)^b^	Range (cm)
MC I	1.58 ± 0.16	1.30–1.70
MC II	2.42 ± 0.13	2.20–2.50
MC III	2.93 ± 0.12	2.80–3.10
MC IV	3.17 ± 0.14	3.00–3.40
MC V	2.65 ± 0.12	2.50–2.80
P1 of digit I	1.20 ± 0.11	1.00–1.30
P1 of digit II	1.70 ± 0.13	1.50–1.80
P1 of digit III	1.98 ± 0.10	1.80–2.10
P1 of digit IV	3.17 ± 0.14	3.00–3.40
P1 of digit V	1.65 ± 0.08	1.60–1.80

*n =* 6.

MC, Metacarpal; P1, proximal phalanx; s.d., standard deviation.

^a^, Measurements of the carpal bones, middle and distal phalanges were not performed owing to their oblique presentation.

^b^,Measurements are not compensated for magnification.

**TABLE 3:** Radiographic measurements of the sesamoid bones in captive red pandas.

Bones	Variables	Mean ± s.d. (cm)^a^	Range (cm)
Sesamoid bone for the abductor digiti I longus	Length	0.32 ± 0.04	0.30–0.40
	ML diameter	0.40 ± 0.06	0.30–0.50
MCP digit I axial	Length	0.35 ± 0.05	0.30–0.40
MCP digit I abaxial	Length	0.32 ± 0.03	0.30–0.35
MCP digit II axial	Length	0.42 ± 0.04	0.40–0.50
MCP digit II abaxial	Length	0.38 ± 0.04	0.30–0.40
MCP digit III axial	Length	0.45 ± 0.05	0.40–0.50
MCP digit III abaxial	Length	0.50 ± 0.06	0.40–0.60
MCP digit IV axial	Length	0.49 ± 0.02	0.45–0.50
MCP digit IV abaxial	Length	0.47 ± 0.04	0.40–0.50
MCP digit V axial	Length	0.56 ± 0.05	0.40–0.50
MCP digit V abaxial	Length	0.43 ± 0.05	0.40–0.50

*n =* 6.

MCP, metacarpophalangeal; ML, mediolateral; s.d., standard deviation.

^a^, Measurements are not compensated for magnification.

### Clavicle

**FIGURE 1:**
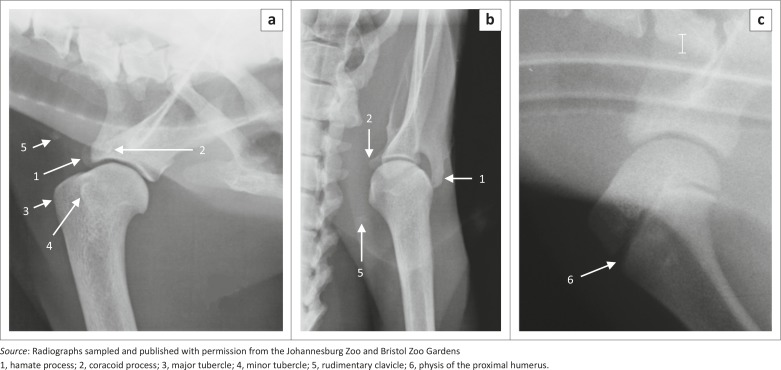
Radiographs of the right shoulder joints of (a, b) a 14.3-year-old male and (c) a skeletally immature red panda. Mediolateral views are shown in (a) and (c) and a caudocranial view in (b).

A rudimentary clavicle was seen in two animals (aged 14.3 and 1.4 years) as an area of mineral opacity in the soft tissues just cranial to the supraglenoid tubercle, as shown on the ML view of the shoulder joint of the older animal (Figure 1a). On the caudocranial (CdCr) view of the shoulder joint, the rudimentary clavicle was seen distomedial to the minor tubercle (Figure 1b) in this animal.

### Scapula

**FIGURE 2:**
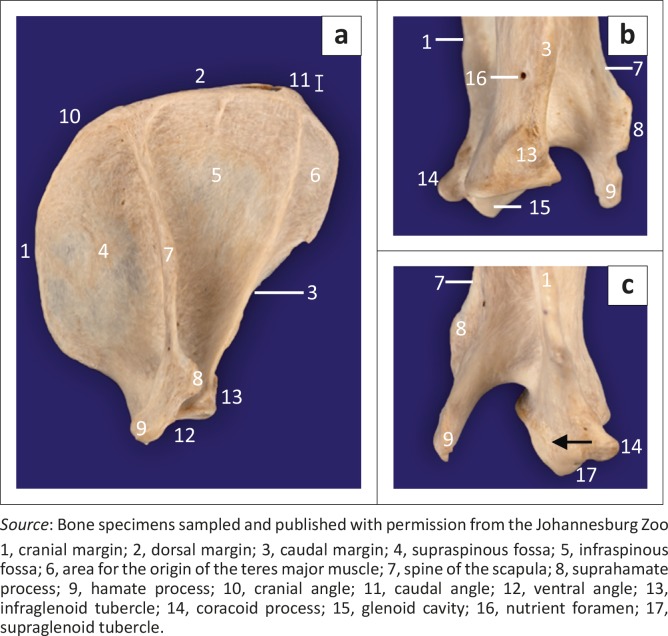
(a) Lateral, (b) caudal and (c) cranial views of a specimen of the right scapula of an adult red panda. Black arrow shows the proximal extension of the articular surface of the glenoid cavity.

The scapula was wide craniocaudally with a convex cranial margin (Figure 2a). The proximal half of the caudal margin was almost convex, whereas the distal half was fairly straight with a rough surface (Figure 2a). The mediodistally directed coracoid process was stout (Figure 2b, c) and the infraglenoid tubercle was well developed (Figure 2b). On the ML view of the shoulder joint (Figure 1a), the coracoid process was seen as a curvilinear area of increased mineral opacity caudal to the supraglenoid tubercle. The articular surface of the glenoid cavity was concave and extended proximal to the lateral area of the supraglenoid tubercle (Figure 2c).

The infraspinous fossa was deep and concave, whereas the supraspinous fossa was convex (Figure 2a). Caudal to the proximal half of the infraspinous fossa, the lateral surface presented a large area for the origin of the teres major muscle (Figure 2a). The spine of the scapula was slanted slightly towards the infraspinous fossa (Figure 2a). The craniodistally directed hamate process was prominently large and flattened mediolaterally (Figure 2a−c). On the ML view of the shoulder joint (Figure 1a), the hamate process projected cranially beyond the cranial margin of the supraglenoid tubercle. On the CdCr view, it projected distally beyond the level of the glenoid cavity (Figure 1b). The medial surface of the scapula presented with a triangular facies serrata.

### Humerus

**FIGURE 3:**
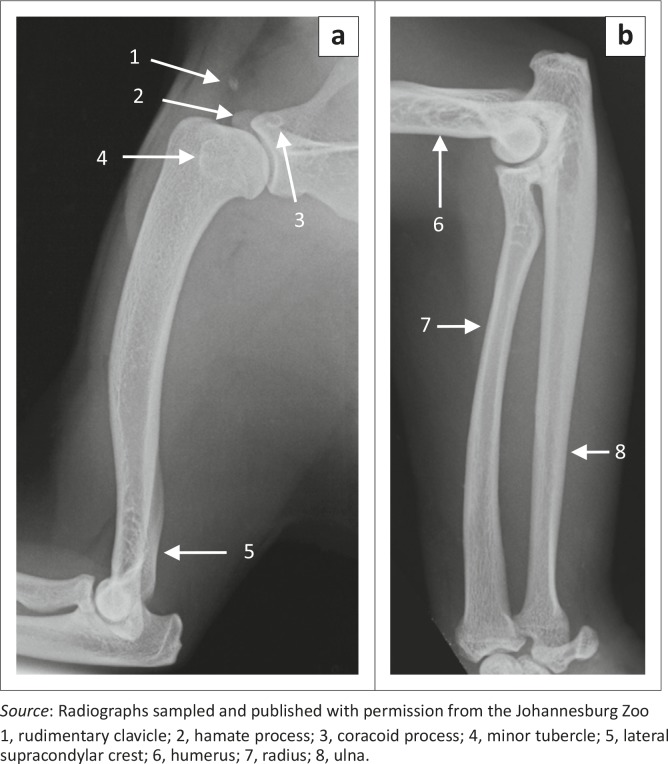
Mediolateral radiographs of (a) the right humerus and (b) the radius and ulna of two male red pandas. Bones of a 1.4-year-old animal are shown in (a) and those of a 2.5-year-old animal are shown in (b).

**FIGURE 4:**
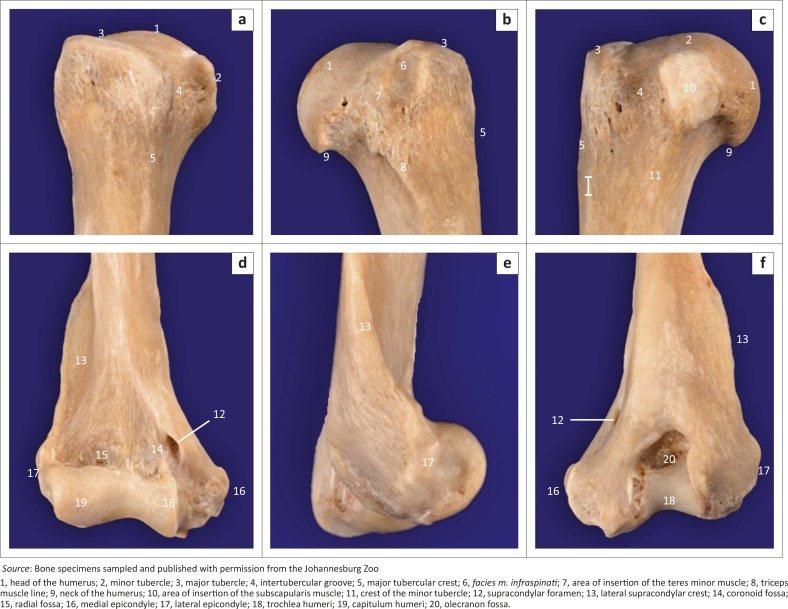
Bone specimen of (a–c) the right proximal and (d–f) the distal humerus of an adult red panda. Cranial views are shown in (a) and (d). Lateral views are shown in (b) and (e). Medial and caudal views are shown in (c) and (f), respectively.

The humerus was the longest bone of the thoracic limb (Table 1). The major and minor tubercles did not extend higher than the head of the humerus (Figure 3a and Figure 4a−c). The major tubercle was almost square, with a flat proximal margin (Figure 4a). When viewed medially, the proximal and cranial margins of the minor tubercle formed an approximately right angle (Figure 4c). On the ML view of the shoulder joint, the major and minor tubercles were further distal than the head of the humerus (Figure 1a). The cranial and proximal margins of the minor tubercle appeared as a square area of increased mineral opacity caudal to the intertubercular groove (Figure 1a). On the CdCr view of the shoulder joint, the major and minor tubercles were fairly flattened (Figure 1b).

The area for the insertion of the infraspinatus muscle (*facies m. infraspinati*) was prominent, ovoid, elongated in a proximodistal direction and presented as a shallow fossa (Figure 4b). It was at the same level as the head of the humerus (Figure 4b). The wide and prominent area for the insertion of the subscapularis muscle faced caudomedially (Figure 4c). The tuberosity for the teres minor muscle was not seen.

**FIGURE 5:**
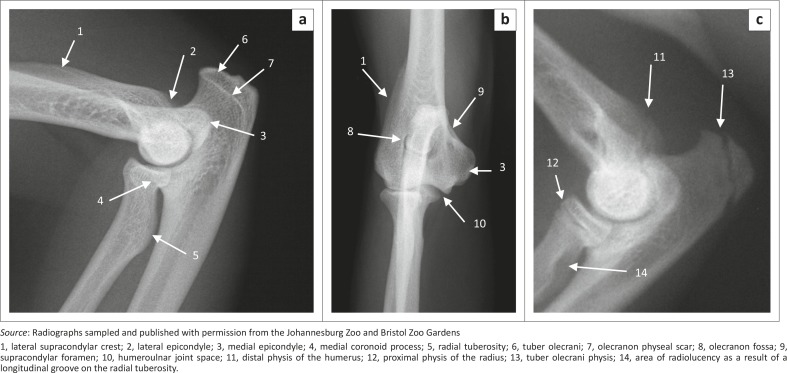
Radiographs of the right elbow joint of (a) a 1.4-year-old, (b) a 10.3-year-old and (c) a skeletally immature male red panda. Mediolateral views are shown in (a) and (c) and a craniocaudal view is shown in (b).

The medial epicondyle was well developed and larger than the lateral epicondyle (Figure 4d, f). The medial and lateral epicondyles presented with shallow depressions for the origin of the flexors and extensors of the carpus and digits, respectively. On the ML view of the elbow joint, the medial epicondyle was square, whereas the lateral epicondyle formed an obtuse angle (Figure 5a). On the CrCd view of the elbow joint, the medial and lateral margins of the medial and lateral epicondyles, respectively, appeared undulating (Figure 5b).

The lateral supracondylar crest was prominent (Figure 4d−f) and projected caudally beyond the cortical bone on the ML view of the elbow joint (Figure 5a). The medially located supracondylar foramen sloped from caudomedial to craniodistal (Figure 4d, f). On the CrCd view of the elbow joint, the supracondylar foramen was represented by two ovoid radiolucent areas just proximal to the medial epicondyle (Figure 5b). The ovoid olecranon fossa sloped from proximolateral to distomedial (Figure 4f and Figure 5b). It was moderately deep and elongated mediolaterally (Figure 4f).

The cranial surface of the condyle of the humerus had well-defined radial and coronoid fossae (Figure 4d). The articular surface of the condyle of the humerus presented with a larger grooved and medially located trochlea humeri and a small laterally located capitulum humeri (Figure 4d). The cranial surface of the trochlea humeri had a prominent medial trochlea lip (Figure 4d), whereas the caudal surface was concave (Figure 4f). The proximal and distal physes of the humerus (Figure 1c and Figure 5c) were similar to those of the domestic cat and dog.

### Radius and ulna

**FIGURE 6:**
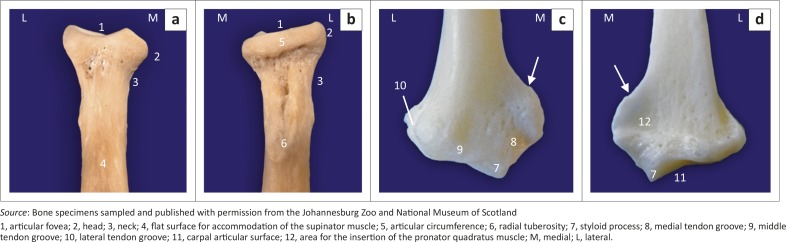
Bone specimens of (a, b) the right proximal and (c, d) the distal radius of adult red pandas. Cranial views are shown in (a) and (c). Caudal views are shown in (b) and (d). White arrows in (c) and (d) indicate a prominent crest for the insertion of the brachioradialis muscle.

The ulna was longer and relatively larger than the radius (Figure 3b; Table 1). The head of the radius sloped from lateral to medial and was elliptical in outline (Figure 6a, b). It had a concave articular fovea (Figure 6a, b) and was demarcated from the body by a distinct neck (Figure 5 and Figure 6). The medial half of the cranial surface of the head of the radius was flat, whereas its lateral counterpart was convex (Figure 6a). The caudal surface of the head of the radius presented with a smooth band, the articular circumference, for articulation with the radial notch of the ulna (Figure 6b).

The body of the radius curved cranially along the longitudinal axis of the bone (Figure 3b). The proximal half of the cranial surface of the body presented with a flat surface (Figure 6a). The radial tuberosity was prominent and located relatively further distally (Figure 5a and Figure 6b). It had a longitudinal groove (Figure 6b). The trochlea of the radius had three cranial grooves for the passage of tendons (Figure 6c). Medially, it presented with a prominent crest (Figure 6c and Figure 6d). The ulnar notch was concave and elongated craniocaudally.

**FIGURE 7:**
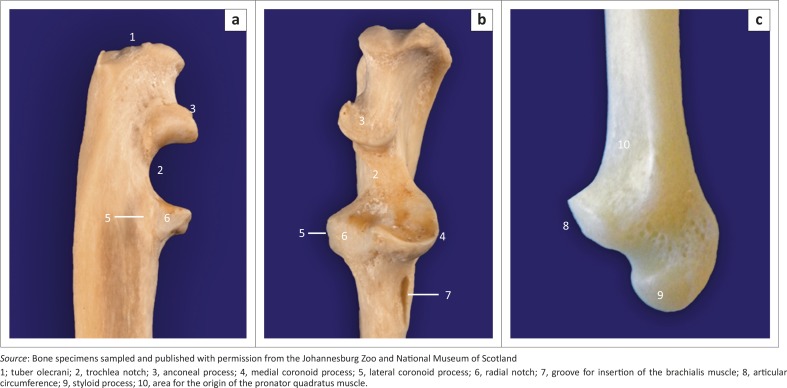
(a) Lateral, (b) cranial and (c) medial views of bone specimens of (a, b) the right proximal and (c) the distal ulna of adult red pandas.

The ulna curved laterally along the longitudinal axis of the bone. The tuber olecrani was square (Figure 5a and Figure 7a). On the ML view of the elbow joint, a thin, straight radiopaque line was seen to run almost parallel with the proximal margin of the tuber olecrani, representing the physeal scar of the tuber olecrani (Figure 5a). The medial coronoid process was prominent and rounded (Figure 5a and Figure 7b). The anconeal process sloped from lateral to medial (Figure 7b), with its lateral margin wrapped proximally against the lateral surface (Figure 7a). The craniolaterally located radial notch was at the same level as the body of the ulna and consisted of a single articular facet (Figure 7a). Just distal to the medial coronoid process, the body of the ulna presented with a deep narrow groove for the insertion of the brachialis muscle (Figure 7a).

**FIGURE 8:**
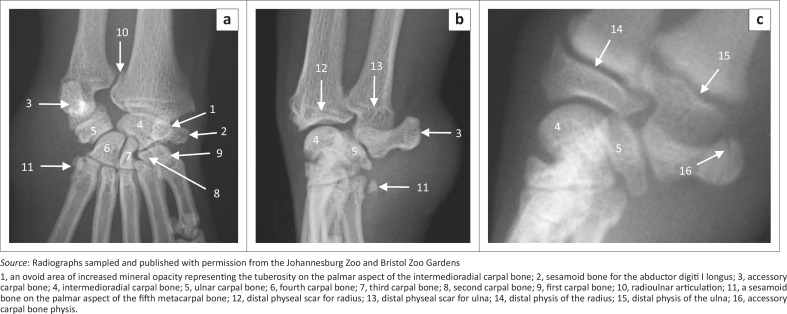
(a) Dorsopalmar and (b, c) mediolateral radiographs of the right carpus of red pandas. The carpus of a 1.4-year-old female animal is shown in (a) and (b), whereas that of a skeletally immature animal is shown in (c).

The head of the ulna was well developed (Figure 7c). The articular circumference was markedly elevated from the body of the ulna and formed almost a right angle with the styloid process (Figure 7c). The styloid process was large and bulbous (Figure 7c). On the ML view of the elbow joint, the physes of the tuber olecrani and proximal part of the radius (Figure 5c) were similar to those of the domestic cat and dog. The physes of the distal part of the radius and ulna appeared transverse (Figure 8c), similar to those of the domestic cat.

### Carpus

The carpus consisted of seven carpal bones: the inter­medioradial, ulnar, accessory, and the first, second, third and fourth carpal bones (Figure 8a). The intermedioradial carpal bone was the largest carpal bone. Palmaromedially it presented with a tuberosity (Figure 8a). Medially the bone presented with an ovoid articular facet for the sesamoid bone of the abductor digiti I longus.

The palmarolaterally directed accessory carpal bone was prominent and almost the same size as the intermedioradial carpal bone (Figure 8a). It had two articular surfaces for the ulnar carpal bone and styloid process of the ulna (Figure 8). On the ML view of the carpus, the physis of the accessory carpal bone appeared sagittal (Figure 8c), similar to that of the domestic cat and dog.

The fourth carpal bone was the largest bone in the distal row (Figure 8a). The third carpal bone was flattened mediolaterally and comma shaped on the DPa view of the carpus (Figure 8a). The second carpal bone was the smallest bone in the distal row and was almost triangular (Figure 8a). The first carpal bone was well developed and articulated distally with the first metacarpal bone (Figure 8a).

### Metacarpus and digits

**FIGURE 9:**
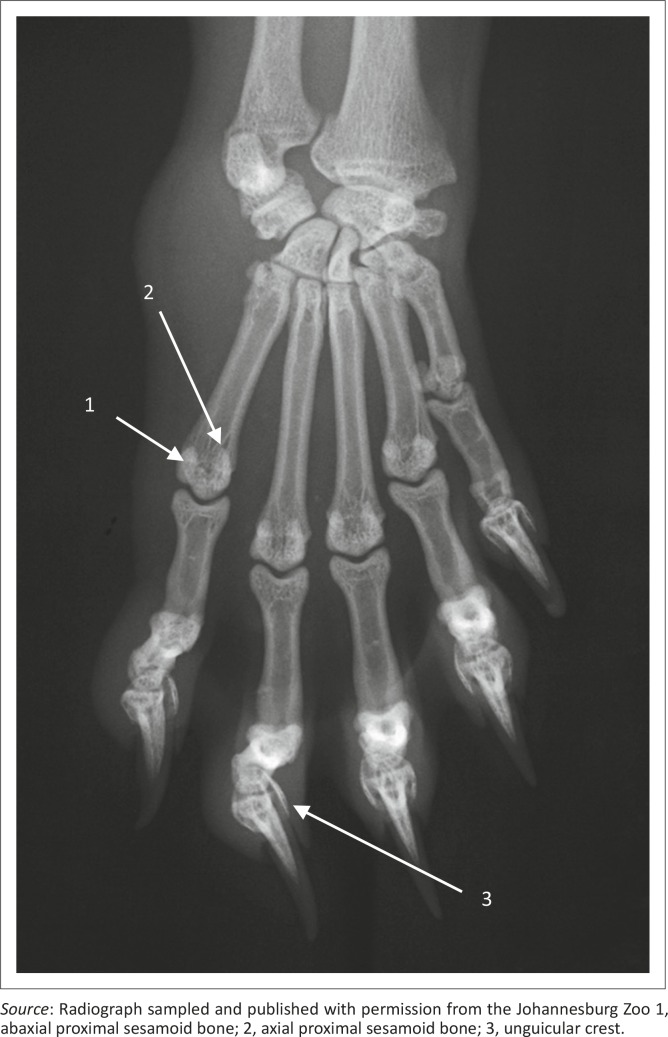
Dorsopalmar radiograph of the right manus of a 1.4-year-old female red panda.

**FIGURE 10:**
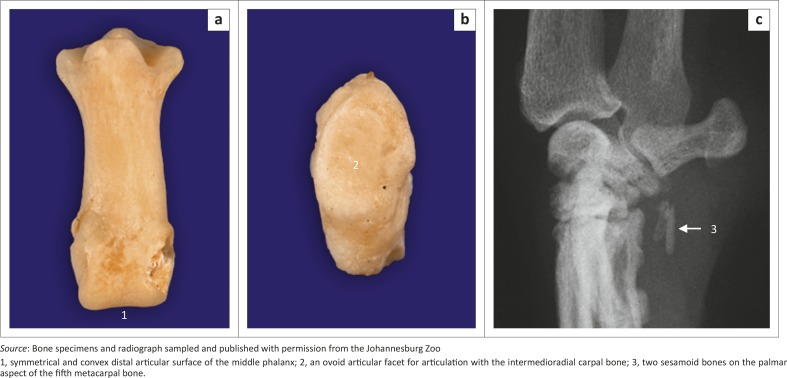
(a) Dorsal and (b) lateral views of bone specimens of the middle phalanx and sesamoid bone for the abductor digiti I longus, respectively, of an adult red panda. (c) Mediolateral radiograph of the right carpus of an 11.3-year-old male red panda.

Five metacarpal (MC) bones and digits were seen (Figure 9). All MC bones were almost equally developed with regard to width (Figure 9). The MC bones were widely spread and relatively short in relation to the length of the digits (Figure 9). The first digit had only two phalanges, namely the proximal and distal phalanges. The other digits (II−V) had three phalanges: proximal, middle and distal phalanges (Figure 9). The distal articular surface of the middle phalanx was symmetrical and convex (Figure 10a). The proximal articular surface of the distal phalanx was concave and parallel to the horizontal plane.

### Sesamoid bones

A large sesamoid bone of the abductor digiti I longus was seen in all animals (Figure 8a and Figure 10b). On the DPa view of the carpus, it was seen medial to the intermedioradial carpal bone (Figure 8a). On the ML view of the carpus, it was not seen owing to its superimposition on the carpal bones (Figure 8b). Each metacarpophalangeal joint had paired proximal sesamoid bones on the palmar aspect (Figure 9).

### Other findings

On the ML view of the carpus, four animals had one bone and two animals had two bones on the palmar aspect of the fifth MC bone, which are likely sesamoid bones (Figure 8b and Figure 10c). (One animal did not have any bones on the palmar aspect of the fifth MC bone.) On the DPa view of the carpus, the bones were superimposed on the abaxial palmar aspect of the fifth MC bone and therefore poorly visible (Figure 8a).

Osteoarthrosis was seen in the elbow of two animals and in the carpus of three animals, all older than nine years. The two animals with elbow osteoarthrosis had concurrent osteoarthrosis of the carpus. Of the two joints, the elbow was more severely affected. Elbow osteoarthrosis was characterised by sclerosis of the trochlea notch, poor visualisation of the medial coronoid process, thickening of the cranial cortex of the distal part of the humerus and the presence of osteophytes dorsally on the anconeal process, the cranial aspect of the humeroradial joint, around the trochlea notch and the lateral epicondylar crest. Osteoarthrosis of the carpus was characterised by poor delineation of the carpal bones, osteophytes on the distal part of the radius and styloid process of the radius and enthesophytes on the lateral and medial margins of the bases of the metacarpal bones, distal part of the sesamoid bone for the abductor digiti I longus, lateral aspect of the fourth carpal bone and ulnar carpal bone.

## Discussion

Less cursorial animals inhabit high-structured habitats such as dense forests, whereas cursorial animals inhabit more open terrain (Gonyea 1978). The thoracic limb of less cursorial animals is capable of a greater range and variety of movements compared with cursorial animals (Hopwood 1947). The difference in movements of the thoracic limb between these two groups of animals is a result of variation in their feeding habits and the environments in which they live (Gonyea 1978; Hopwood 1947). In the red panda, movement on and between the small terminal branches of trees is facilitated by a high degree of flexibility of the pectoral and pelvic girdles and limb joints (Roberts & Gittleman 1984).

The wide scapula observed in the red panda accommodates well-developed muscles, which stabilise the shoulder joint and prevent luxation. Fisher *et al.* (2009) reported stout tendons of insertion of the supraspinatus and infraspinatus muscles, which stabilise the shoulder joint in the red panda. In arboreal quadrupedal animals, the thoracic limb is frequently used in an abducted position and therefore the muscles that stabilise the shoulder joint are well developed (Roberts & Davidson 1975).

The presence of a prominent, large hamate process, which provides the origin of the deltoideus pars acromialis (Fisher *et al.* 2009), and the location of the insertion of the infraspinatus muscle at the same level as the head of the humerus, enhance the abduction function of the shoulder joint in the red panda. Similar findings have also been reported in some African mammals (Hopwood 1947; Taylor 1974).

The presence of the large area for the origin of the teres major muscle, well-developed medial epicondyle, deep groove for the insertion of the brachialis muscle and prominent further distally located radial tuberosity observed in the red panda indicate the presence of strong flexor muscles of the thoracic limb. Strong flexor muscles of the thoracic limb are important in arboreal quadrupeds as the thoracic limb is frequently held in a flexed position during locomotion (Hopwood 1947). The teres major and brachialis muscles flex the shoulder and elbow joints, respectively (Fisher *et al.* 2009). The medial epicondyle provides the origin for the flexor muscles of the carpus and digits (Fisher *et al.* 2009). The radial tuberosity provides the insertion for the biceps brachii, which flexes the elbow joint (Fisher *et al.* 2009). The radial tuberosity was also found to be larger and located further distally in the African palm civet (*Nandinia binotata*), a climbing species, which points to the strength of the biceps brachii in flexing the elbow joint (Taylor 1974).

The concave caudal surface of the trochlea humeri in the red panda increases the stability of the elbow joint when the joint is in a flexed position (Fleagle & Simons 1995). The prominent medial trochlea lip counteracts the adducting forces at the elbow joint produced by the carpal and digital flexors when the antebrachium and manus are in a pronated position (Fleagle & Simons 1995).

The oblique orientation of the olecranon fossa observed in the red panda, sloping from proximolateral to distomedial, indicates that the thoracic limb moves through an arch during locomotion, away from the parasagittal plane of the body, as in most feline species (Gonyea 1978). This phenomenon most likely reflects an adaptation to the arboreal environment in which it lives. In felids, it was found that species that are exclusive forest dwellers have a greater angle of inclination of the olecranon fossa compared with those that inhabit more open terrain (Gonyea 1978).

The power to rotate the antebrachium, which is of great importance to a climbing species (Hopwood 1947), is also important because the manus is used as a prehensile organ for food manipulation tasks such as grasping and bending bamboo to bring the leaves within reach of the mouth (Antón *et al.* 2006; Roberts & Gittleman 1984). In the red panda, the power to rotate the antebrachium was indicated by the presence of the following features: the elliptically shaped head of the radius, stout and well-developed body and head of the ulna, well-developed muscles of supination and a craniolaterally located radial notch with a single articular facet at the same level as the body of the ulna (Gonyea 1978; Taylor 1974).

During pronation and supination, the elliptically shaped head of the radius acts as a cam, which imparts the eccentric motion of the radius (Gonyea 1978). The stout and well-developed body and head of the ulna act as a pivot, which supports the radius during pronation and supination. The prominent lateral supracondylar crest provides the origin for the brachioradialis muscle, which supinates the antebrachium (Fisher *et al.* 2009). A distinct flattening on the proximal half of the cranial surface of the radius accommodates the supinator muscle, which supinates the antebrachium (Fisher *et al.* 2009). In addition, the abductor digiti I longus assists in supination (Antón *et al.* 2006). The deep-sited, cranially located radial notch, with two articular facets, restricts rotation of the radius (Gonyea 1978; Taylor 1974).

The palmarolaterally directed accessory carpal bone, large sesamoid bone for the abductor digiti I longus and widely spread MC bones indicate flexibility of the manus in the red panda, which is important for arboreal locomotion and food manipulation (Antón *et al.* 2006; Taylor 1974). The palmarolaterally directed accessory carpal bone enables greater leverage to be exerted on the manus during ulnar deviation (Taylor 1974). A large sesamoid bone for the abductor digiti I longus provides the insertion of the muscle, which abducts the carpal joint and carpometacarpal joint of digit I (Fisher *et al.* 2009) and also supinates the manus (Antón *et al.* 2006).

In addition, the sesamoid bone of the abductor digiti I longus acts as a ‘false-thumb’ for the grasping actions of the manus (Antón *et al.* 2006). Widely spread and relatively short MC bones provide a wide space and allow greater effectiveness of the muscles responsible for small movements of the digits (Taylor 1974). The paired proximal sesamoid bones provide insertions for a lateral and medial belly of flexores breves profundi muscles (Fisher *et al.* 2009). These muscles flex the metacarpophalangeal joints and may abduct or adduct the digits (Fisher *et al.* 2009).

The presence of osteoarthrosis in animals older than nine years in this study, without medical history of trauma, suggests that the likely cause is primary osteoarthrosis. It is likely that these two joints are subjected to more stress as a result of arboreal locomotion; however, this warrants further investigation. A review of feline osteoarthrosis indicated that for a large proportion of animals presenting with osteoarthrosis, there was no obvious cause, suggesting a primary osteoarthrosis (Lascelles 2010).

The small bones located on the palmar aspect of MC V are likely sesamoid bones. However, in this study it was difficult to ascertain which muscle they are associated with. The symmetrical shape of the distal articular surface of the middle phalanx and the parallel orientation to the horizontal plane of the proximal articular surface of the distal phalanx observed in this study are different from felids’ (Gonyea & Ashworth 1975) and indicate that the claws of the red panda are slightly retractile (Taylor 1974) or not able to retract at all (Gonyea & Ashworth 1975).

## Conclusion

Morphology of the thoracic limb of the red panda evidenced by osteology and radiography indicated flexibility of the thoracic limb joints and well-developed flexor and supinator muscles, which are important in arboreal quadrupedal locomotion. Knowledge of the normal osteology and radiographic anatomy of the thoracic limb of the red panda may prove useful in identifying skeletal material or remains and diagnosis of musculoskeletal diseases and injuries of the thoracic limb.

## References

[CIT0001] Antón M., Salesa M.J., Pator J.F., Peigné S. & Morales J., 2006, ‘Implication of the functional anatomy of the hand and forearm of *Ailurus fulgens* (Carnivora, Ailuridae) for the evolution of the ‘false-thumb’ in pandas’, *Journal of Anatomy* 209(6), 757−764 10.1111/j.1469-7580.2006.00649.x 17118063 PMC2049003

[CIT0002] Carlsson A., 1925, ‘Über Ailurus fulgens [About Ailurus fulgens]’, Acta Zoologica 6(1−2), 269−305 10.1111/j.1463-6395.1925.tb00268.x

[CIT0003] Fisher R.E., Adrian B., Barton M., Holmgren J. & Tang S.Y., 2009, ‘The phylogeny of the red panda (Ailurus fulgens): Evidence from the forelimb’, *Journal of Anatomy* 215(6), 611−635 10.1111/j.1469-7580.2009.01156.x 19930516 PMC2796785

[CIT0004] Fleagle J.G. & Simons E.L., 1995, ‘Limb skeleton and locomotor adaptations of Apidium phiomense, an Oligocene anthropoid from Egypt’, *American Journal of Physical Anthropology* 97(3), 235−289 10.1002/ajpa.1330970303 7573376

[CIT0005] Gonyea W.J., 1978, ‘Functional implications of felid forelimb anatomy’, *Acta Anatomica* 102(2), 111−121 10.1159/000145627 685643

[CIT0006] Gonyea W. & Ashworth R., 1975, ‘The form and function of retractile claws in the felidae and other representative carnivorans’, *Journal of Morphology* 145(2), 229−238 10.1002/jmor.1051450208 1127699

[CIT0007] Hopwood A.T., 1947, ‘Contributions to the study of some African mammals III. Adaptations on the bones of the forelimb of the lion, leopard and cheetah’, *Journal of the Linnean Society of London, Zoology* 41(279), 259−271.

[CIT0008] IUCN, 2014, ‘Ailurus fulgens (Red Panda)’, in *IUCN Red List of Threatened Species*, version 2014 3, viewed 10 March 2015, from http://www.iucnredlist.org

[CIT0009] Lascelles B.D.X., 2010, ‘Feline degenerative joint disease’, *Veterinary Surgery* 39(1), 2−13 10.1111/j.1532-950X.2009.00597.x 20210938

[CIT0010] Lynch M., McCracken H. & Slocombe R., 2002, ‘Hyperostotic bone disease in red pandas (Ailurus fulgens)’, *Journal of Zoo and Wildlife* 33(3), 263−271 10.1638/1042-7260(2002)033[0263:HBDIRP]2.0.CO;2 12462494

[CIT0011] Nickel R., Schummer A., Seiferle E., Frewein J., Wilkens H. & Wille K.H., 1986, *The anatomy of the domestic animals*, Paul Parey, Berlin.

[CIT0012] Philippa J. & Ramsay E., 2011, ‘Captive red panda medicine’, in Glatston A.R. (ed.), *Red panda biology and conservation of the first panda*, pp. 271−285, Academic Press, London 10.1016/b978-1-4377-7813-7.00015-x

[CIT0013] Preece B., 2011, ‘Red panda pathology’, in Glatston A.R. (ed.), *Red panda biology and conservation of the first panda*, pp. 287−302, Academic Press, London 10.1016/b978-1-4377-7813-7.00016-1

[CIT0014] Roberts D. & Davidson I., 1975, ‘The lemur scapula’, in Tattersall I. & Sussman R.W. (eds.), *Lemur Biology*, pp. 125−147, Plenum Press, New York 10.1007/978-1-4684-2121-7_8

[CIT0015] Roberts M.S. & Gittleman J.L., 1984, ‘Ailurus fulgens’, *Mammalian Species* 222, 1−8 10.2307/3503840

[CIT0016] Smallwood J.E. & Spaulding K.A., 2013, ‘Radiographic anatomy of the appendicular skeleton’, in Thrall D.E. (ed.), *Textbook of veterinary diagnostic radiology*, 6th edn, pp. 224−251, Saunders Elsevier, St. Louis.

[CIT0017] Taylor M.E., 1974, ‘The functional anatomy of the forelimb of some African Viverridae (Carnivora)’, *Journal of Morphology* 143(3), 307−335 10.1002/jmor.1051430305 4837745

[CIT0018] Thrall D.E. & Robertson I.D., 2011, *Atlas of normal radiographic anatomy and anatomic variants in the dog and cat*, Elsevier Saunders, St. Louis.

